# Mechanism-based organization of neural networks to emulate systems biology and pharmacology models

**DOI:** 10.1038/s41598-024-59378-9

**Published:** 2024-05-27

**Authors:** John Mann, Hamed Meshkin, Joel Zirkle, Xiaomei Han, Bradlee Thrasher, Anik Chaturbedi, Ghazal Arabidarrehdor, Zhihua Li

**Affiliations:** Division of Applied Regulatory Science, Office of Clinical Pharmacology, Office of Translational Sciences, Center for Drug Evaluation and Research, U.S. Food and Drug Administration, WO Bldg 64 Rm 2084, 10903 New Hampshire Ave, Silver Spring, MD 20993 USA

**Keywords:** Public health, Computational biology and bioinformatics, Systems biology, Mathematics and computing

## Abstract

Deep learning neural networks are often described as black boxes, as it is difficult to trace model outputs back to model inputs due to a lack of clarity over the internal mechanisms. This is even true for those neural networks designed to emulate mechanistic models, which simply learn a mapping between the inputs and outputs of mechanistic models, ignoring the underlying processes. Using a mechanistic model studying the pharmacological interaction between opioids and naloxone as a proof-of-concept example, we demonstrated that by reorganizing the neural networks’ layers to mimic the structure of the mechanistic model, it is possible to achieve better training rates and prediction accuracy relative to the previously proposed black-box neural networks, while maintaining the interpretability of the mechanistic simulations. Our framework can be used to emulate mechanistic models in a large parameter space and offers an example on the utility of increasing the interpretability of deep learning networks.

## Introduction

Machine learning models are a subset of artificial intelligence (AI) that utilize algorithms to imitate human-like learning and intelligence^1^. These types of models are increasingly being used to solve complex problems across all areas of research including the development of autonomous vehicles, superhuman mastery of chess or go, or even advertising and marketing^2,3^ The healthcare space is no exception, with machine learning models being employed for natural language processing (NLP) of COVID-19 research findings, in silico simulation of massive clinical trials, and even the discovery and development of new drug formulations^4–6^. In recent years, AI has become widely adopted and even commonplace within the healthcare and regulatory spaces with over 500 machine learning applications being approved as Software as a Medical Device (SaMD) by FDA to date. The 2021 FDA AIML SaMD Action Plan further cements the expansion of AI applications into modern health care and regulation^7,8^.

While these AI tools are widely used and allow for rapid results and promising research breakthroughs, they are often viewed as “black boxes,” wherein it is difficult to trace model outputs back to model inputs due to a lack of clarity over the internal mechanisms. This ambiguity has led to calls to find better methods to explain AI outputs or to even do away with these types of models entirely in favor of more understandable alternatives for high impact decision making^9,10^. This presents a unique and challenging dilemma with model utility being pitted against user and public confidence.

One particularly interesting example highlighting this mechanism vs. black-box dilemma is the use of deep learning neural networks to emulate mechanistic model simulations^11–13^. For instance, systems biology or pharmacology models are typically mechanistic models using mathematical equations to quantitatively describe essential biological or pharmacological processes underlying the systems dynamics (time courses of physiological changes or pharmacological measurements). As numerical simulation of these equations is time consuming, recently Wang et al. proposed an artificial neural networks-based method that can learn a mapping between the parameters of mechanistic models and the final systems dynamics, bypassing the underlying mechanisms completely^14^. While demonstrating massive acceleration in computational speed, this method “flips” a mechanistic model into a black-box one, trading the former’s strength (transparency and interpretability) for that of the latter (computing efficiency).

In this work, we employed the algorithms proposed by Wang et al.^14^ and endeavored to develop a mechanistically inspired deep learning model capable of leveraging the medium’s strengths without sacrificing interpretability. We found that, by reorganizing the layers of artificial neural networks to mimic the biological/pharmacological processes underlying the systems of interest, it is possible to turn a black-box deep learning model into a semi-mechanistic one. The resulting model not only maintained the clarity of the mechanistic simulations, but also improved training rates and predictive capabilities relative to the previously proposed black-box AI-based emulation approach.

## Methods

### Mechanistic model to simulate respiratory depression under opioid agonists and antagonists

**Figure 1:**
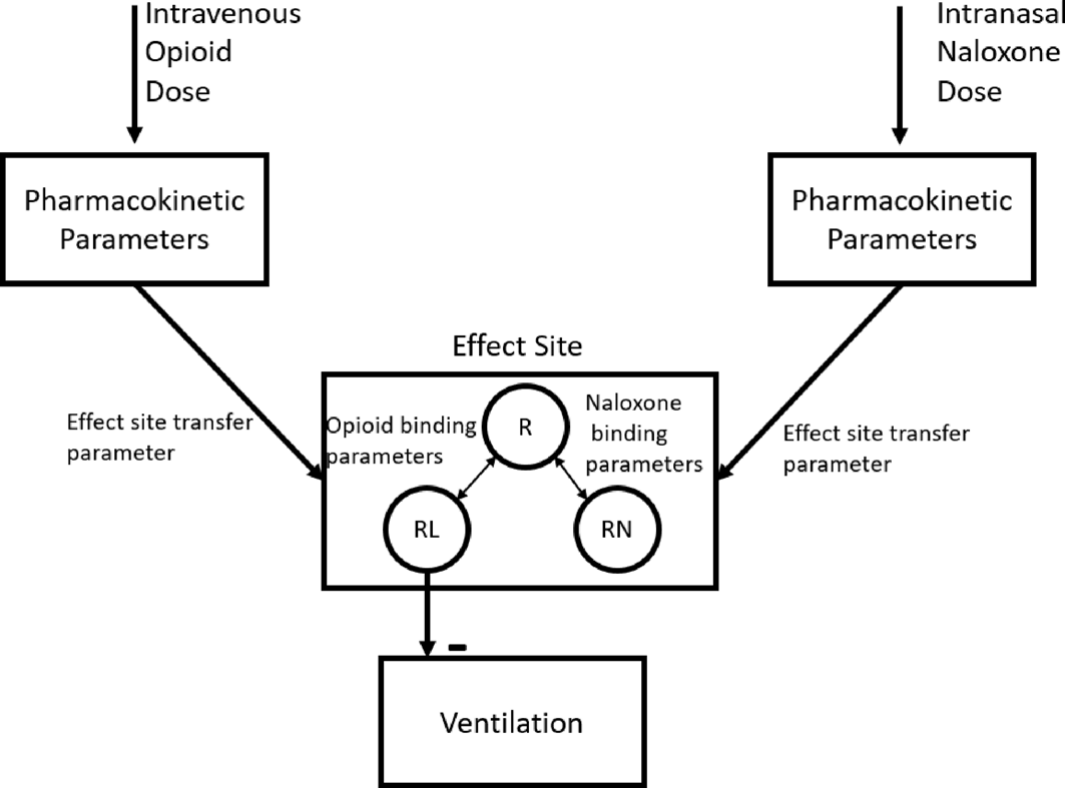
Structure of simplified opioid pharmacokinetic-pharmacodynamic (PK-PD) Model. The model has 3 primary components. The PK component converts doses of either opioid or naloxone, through intravenous and intranasal dosing routes respectively, to their free concentrations in the effective compartment in the brain. The receptor component allows free opioid and naloxone in the brain-effect region to compete for occupancy of the mu opioid receptor. The PD component controls the reduction in fractional minute ventilation by way of the opioid-bound receptor fraction.

Our research group recently developed a translational pharmacokinetic-pharmacodynamic (PK-PD) model for the prediction of opioid overdose and subsequent recovery of respiratory depression after administration of the opioid antagonist naloxone^15^. As a proof of concept, we implemented a simplified version of this model (Fig. 1). This model has sufficient mechanistic information for us to investigate ways to introduce system mechanisms into a deep learning framework. On its own the mechanistic model is specialized in simulating a specific clinical situation where subjects have their alveolar (end-tidal) CO2 partial pressure maintained at an elevated and constant level, a common practice in clinical studies investigating respiratory depression^16–18^. This model has different mechanistic components to describe different biological and pharmacological processes, including receptor binding, PK, and PD. These components work together to determine the dynamics of the clinical variable of interest: the fractional change of minute ventilation volume (*V*_*F*_) under the influence of opioids and naloxone.

The receptor binding component uses the following ordinary differential equation (ODE) to describe the system:

1 $$\frac{d{R}_{L}}{dt}={K}_{on}{L}^{n}R-{K}_{off}{R}_{L}$$

where *L*, *R*, and *R*_*L*_ are free ligands (opioids or naloxone), fraction of free (unoccupied) opioid receptors, and fraction of ligand-occupied receptors, respectively. *K*_*on*_, *K*_*off*_, and *n* are the association (binding) rate, dissociation (unbinding) rate, and the slope of the dose–effect relationship, respectively. For each ligand, these binding parameters were estimated by fitting to in vitro binding data, during which bootstrapping was used to capture the variability of in vitro data and uncertainty of model fitting, resulting in 2000 parameter sets that approximate the joint probability distribution of *K*_*on*_, *K*_*off*_, and *n*^15^.

For the PK component of naloxone, the following equations are used.

2 $$\frac{d{T}_{1}}{dt}={K}_{tr}DF{e}^{-{K}_{tr}t}-{K}_{tr}{T}_{1}$$

3 $$\frac{d{T}_{2}}{dt}={K}_{tr}{T}_{1}-{K}_{in}{T}_{2}$$

4 $$\frac{dP}{dt}=\frac{{K}_{in}}{V}{T}_{2}-\frac{{C}_{L}}{V}P$$

This PK component is a transit compartment model with 2 transition (*T*_*1*_ and *T*_*2*_) and 1 central (*P*) compartment to simulate the delayed absorption of naloxone into the plasma following intranasal (IN) administration. *D* is the drug dose in mg. The parameters *K*_*tr*_, *K*_*in*_,* V*, and *C*_*L*_ (transition rate constant, absorption rate constant, volume of distribution and total clearance respectively) were estimated by fitting to plasma concentration data from the FDA label for NARCAN^19^.

For the PK component of opioids, we used a fentanyl PK model from literature^18^. For the purposes of this case study, carfentanil PK was assumed to match that of fentanyl.

5 $$\frac{d{P}_{F}}{dt}={K}_{21}{P}_{F2}+{K}_{31}{P}_{F3}-{K}_{12}{P}_{F}-{K}_{13}{P}_{F}-{K}_{out}{P}_{F}$$

6 $$\frac{d{P}_{F2}}{dt}={-K}_{21}{P}_{F2}+{K}_{12}{P}_{F}$$

7 $$\frac{d{P}_{F3}}{dt}=-{K}_{31}{P}_{F3}+{K}_{13}{P}_{F}$$

The opioid PK component is a 3-compartment model with 1 central compartment (*P*_*F*_) and 2 peripheral compartments (*P*_*F2*_ and *P*_*F3*_) to simulate bolus administration of IV opioid. The parameters *K*_*out*_, *K*_*12*_*, K*_*21*_, *K*_*13*_, and *K*_*31*_ (elimination rate constant, forward and reverse rate constant between the central and first peripheral compartment, and the forward and reverse rate constant between the central and the second peripheral compartment) were taken from literature where the reported mean and standard deviation were used to sample 2000 parameter sets that approximate the distribution of the PK parameters in a general population with inter-subject variabilities^18^.

For the PD component, the transfer of carfentanil and naloxone from the plasma to the brain effect site was modeled as a biophase transition model with equilibration parameters taken from the literature^18,20^.

8 $$\frac{dL}{dt}=\frac{{k}_{1}{P}_{F}}{{V}_{c}{M}_{mass}}1e9-{k}_{1}L$$

The biophase transition model controls the rate at which the effect site concentration (*L*) equilibriates with the plasma compartment (*P*_*F*_). The parameters *k*_*1*_ and *V*_*c*_ (biophase equilibriation term and central compartment volume) are taken from literature^18,20^ and the 1e9 scaling is used to convert to the pMol concentrations used to estimate the receptor binding parameters.

The effect site concentrations for opioids and naloxone were used as input to the receptor binding component to calculate the fraction of opioid mu receptor occupied by opioids (*R*_*L*_ in Eq. 1), which is then translated into the fraction of minute ventilation volume relative to the baseline:

9 $${V}_{F}=1- \alpha {R}_{L}$$

where *V*_*F*_ is fractional minute ventilation volume, α is the opioid agonism coefficient and R_L_ is fraction opioid receptor occupancy. For fentanyl and its derivatives like carfentanil the *α* value is set to 1^16^.

### Black-box deep learning model as proposed by Wang et al.

**Figure 2:**
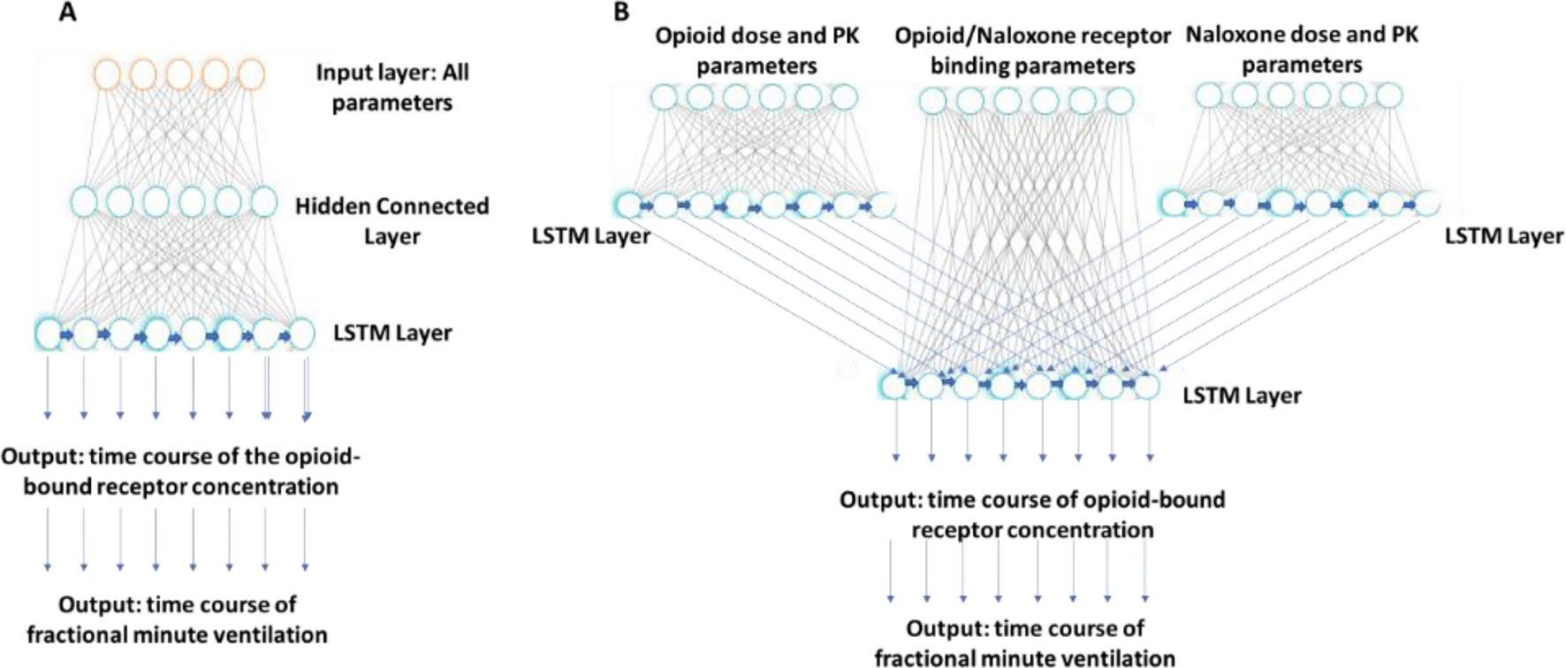
Comparison between Application of the Deep Learning Model Structure in Wang et al. (the black-box model) to Opioid Overdose Simulations and the Mechanistically Inspired Model (the semi-mechanistic model). (**A**): The black-box model is comprised of three layers. The input layer is where all model parameters (opioid and naloxone PK and dosing information as well as receptor binding parameters) are presented to the network. The hidden layer is a fully connected layer. The LSTM layer is where time course data is outputted, in this case: fractional opioid receptor occupancy. (**B**): The semi-mechanistic model splits the input layer into three distinct sub-layers: Opioid dosing and PK parameters, naloxone dosing and PK parameters, and opioid plus naloxone receptor binding parameters. The dosing and PK parameter input layers for opioid and naloxone have their own intermediate LSTM layers to allow for collection of effect-site time course data in addition to the opioid-bound receptor time course data in the final LSTM layer. The structure of the semi-mechanistic model resembles that of the mechanistic model in Fig. 1.

The deep learning model as proposed by Wang et al.^14^ is a Recurrent Neural Network (RNN) utilizing a long short-term memory (LSTM) framework^20^. RNNs are a type of deep learning model that incorporate loops to allow prior states to inform future outputs in time series data. LSTM models are a subset of these RNNs which utilize memory cells to prevent state effects from vanishing over time. Wang et al. proposed to stack fully connected layers, which are widely used as hidden layers for different deep learning tasks, on top of LSTM layers as the internal network structure of their neural network model to emulate mechanistic models. Because the target system’s mechanisms are ignored, the same deep learning structure can be applied to very different mechanistic models (and hence we refer to this type of model as a “black-box” model). We developed such a black-box model similar to Wang et al., which is comprised of a single input layer to receive parameters of the mechanistic model, a hidden fully connected layer, and a LSTM layer for output of opioid receptor occupancy time course, which is then translated into the dynamics of minute ventilation through the PD equation above (Fig. 2(A)).

### Semi-mechanistic deep learning model

The mechanistically inspired machine learning model attempts to mirror the structure of the mechanistic model to better replicate its results. Rather than a single input layer containing all parameters of interest, there are now three distinct input layers: the first for the opioid dose and PK parameters, the second for naloxone dose and PK parameters, and the third for opioid and naloxone receptor binding parameters.

The PK parameters and dosing information for opioids and naloxone both pass to their own middle LSTM layers, which generate internal recurrent data that can be thought of corresponding to the time course of opioid and naloxone’s effect-site concentration in the brain, similar to the mechanistic model. This information is then passed to the final LSTM layer along with the opioid and naloxone receptor binding parameters to produce time course data for the opioid receptor occupancy, followed by translation into minute ventilation. Unlike the black-box model, there are no hidden layers in the semi-mechanistic model. The model structure can be found in Fig. 2(B).

### Training

We trained both the black-box and semi-mechanistic deep learning models based on the inputs and outputs of the mechanistic model. The output is the time course of the mu opioid receptor occupancy following a specific opioid (carfentanil) and naloxone dosing scenario. The inputs include kinetic parameters associated with the mechanistic model, as well as parameters associated with dosing scenarios. For the former, 2000 sets of kinetic parameters were randomly sampled and combined from the probability distributions of PK and receptor binding parameters as estimated through experimental data (see previous sections). For the latter, it includes the opioid dose (12 discrete levels from 0.013 to 0.157 mg), the total number of naloxone doses administered (0, 1, 2, 3, or 4), the respiratory thresholds required to administer naloxone (40%, 25% and 10% of baseline minute ventilation), and the delay between the first and subsequent doses of naloxone for scenarios where additional doses were administered (2, 3 or, 5 min). In total, the 2000 kinetic parameter sets (virtual subjects) and the 540 dosing scenarios led to 1,080,000 parameter combinations as training data. We utilized the same training methodology for both machine learning models with the objective function aiming to minimize the mean square error of opioid receptor occupancy relative to simulated results. As in the publication by Wang et al., we utilized the Adam algorithm of gradient descent to optimize the results^14^. Both models were trained for 48 h on GPUs (NVIDIA Tesla V100 GPU) linked to the FDA’s high-performance computing (HPC) cluster. In each epoch, we randomly set aside 10% of the training data to calculate and report the training error.

### Prediction

The PK and receptor binding parameter distributions were randomly sampled and combined again to generate another set of 2000 kinetic parameters (a new virtual population that is different from the one used in training). The same 540 dosing scenarios were applied, leading to 1,080,000 new parameter combinations as testing samples for both deep learning models to predict. To evaluate the performance of the semi-mechanistic and black-box deep learning models we calculated the overall root mean squared error of the median and 95% confidence intervals of the fractional minute ventilation data against the original mechanistic simulations.

As a predictive “baseline”, we also implemented the Partial Lease Square Regression (PLSR) model using the Scikit-learn library in Python^21^. During training, a 15-fold cross-validation was used to determine the optimal number of PLS components. Subsequently, the trained model was used to predict the outcome of the same 1,080,000 parameter combinations as the black-box and semi-mechanistic AI models.

### Computational systems

The mechanistic model was numerically solved by deSolve in R, a high-level language with a performance similar to MATLAB^22,23^, which was used by Wang et al. to implement their mechanistic models for benchmarking^14^. The deep learning models were implemented in python 3.6 with TensorFlow 1.9^24^. As the computational efficiency depends on the computing resources (e.g., number of CPU or GPUs), we report the normalized time it would take for a single CPU (Intel® Xeon® Gold 6226 CPU @ 2.70GH) to finish the mechanistic model simulation, or a single GPU (NVIDIA Tesla V100 GPU) to finish the neural network computation. To finish one dosing scenario for 2000 virtual subjects, it would take 30 min for the mechanistic model, and 2–3 min for the neural networks. To finish all 540 dosing scenarios on the 2000 virtual subjects, it would take more than 10 days for the mechanistic model, while 19 min for the neural networks.

This study used the computational resources of the High-Performance Computing clusters at the Food and Drug Administration, Center for Devices and Radiological Health.

## Results

### The conceptual framework of reorganizing deep learning neural networks to mimic the mechanisms of the target systems

The structure of a mechanistic model can usually by depicted as a diagram to give a conceptual presentation of the underlying processes (mechanisms) of the target system. For example, a pharmacokinetic-pharmacodynamic (PK-PD) model about the effects of opioids and naloxone on respiratory depression, such as the recently published translational model^15^, could have processes depicting the accumulation and clearance of opioids and naloxone in the human body, the competition between opioids and naloxone in binding to the opioid receptor, and the effects of opioid-bound receptor on human’s ventilation volume per minute (minute ventilation or MV) as a clinical endpoint. Such a mechanistic model could be depicted as a diagram in Fig. 1.

In contrast, although conceptually inspired by the human brain^25^ , typical artificial neural networks differ significantly from biological neural networks^26^ on the structural or mechanistic level. This is even true when the deep learning model was designed to emulate a specific biological system. For example, Wang et al. recently proposed a deep learning model based on Long-Short-Term Memory (LSTM) units that can be trained by a relatively small number of simulations generated by a mechanistic model, and subsequently used in place of the mechanistic model to simulate the target system in a larger parametric space and under more scenarios^14^. While there is a significant gain in computational speed with such an approach, the deep learning neural networks would lose all mechanistic information about the target system and become a “black-box” as it is hard to trace the output back to the input. A neural network similar to Wang et al. for emulating the PK-PD model above is shown in Fig. 2(A).

A comparison between the mechanistic PK-PD model (Fig. 1) and the black-box deep learning model (Fig. 2(A)) reveals distinct structural differences. For example, in the black-box model, the information contained in the kinetic parameters of different sub-processes (PK, receptor binding, etc.), as well as the information contained in the parameters about the overdose scenarios (opioid dose, naloxone dose, etc.) are all propagated into the common hidden layer (Fig. 2(A)). In contrast, in the mechanistic model, these different types of information were segregated into different components, and only merged in the final step, when the PK and receptor binding components are connected (Fig. 1). We reorganized the layers of neural networks to better mimic the structure of the mechanistic model. In this new model (Fig. 2(B)), the information flow is divided into three parts: the PK and dose of opioids is connected to one LSTM layer to mimic the opioids PK component; the PK and dose of naloxone is connected to another LSTM layer to mimic the naloxone PK component; and the outputs from the two LSTM layers above are combined with the opioid and naloxone receptor binding parameters to mimic the connection of the PK and receptor binding components in the mechanistic model. We call such a model a “semi-mechanistic deep learning model” as it is a deep learning framework with the neural network structure reorganized to partially mimic the target system it tries to emulate.

### The semi-mechanistic deep learning model outperforms the black-box model in training

**Figure 3:**
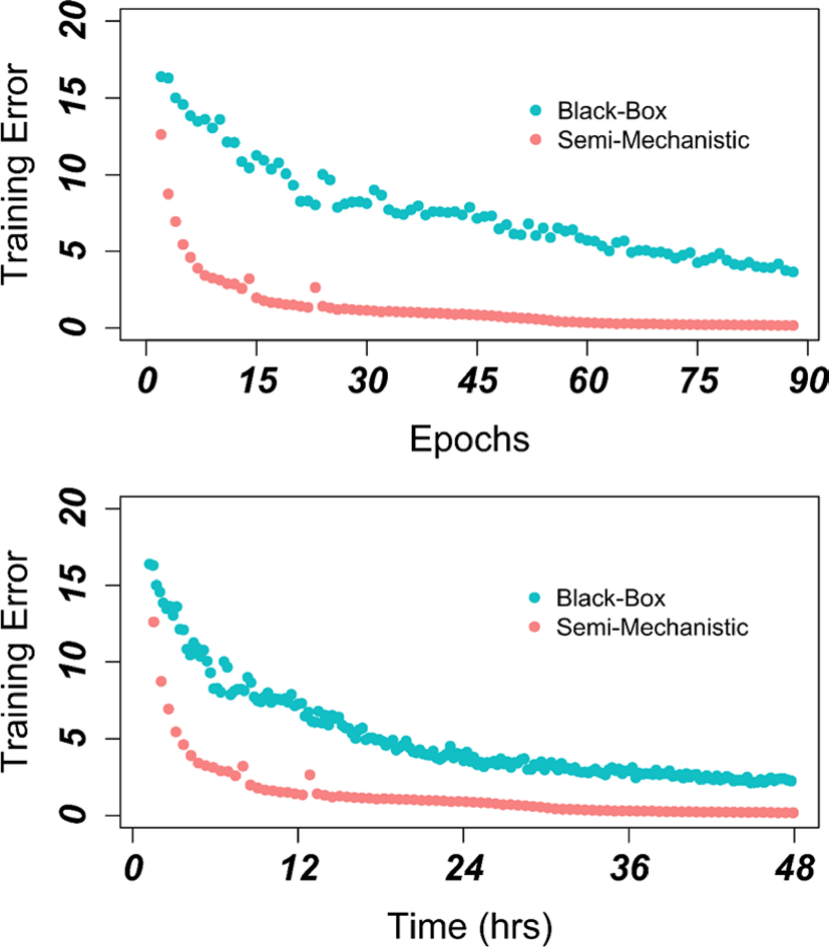
Rate of Training Error Improvement in the Black Box and Semi-mechanistic Deep Learning Models. The semi-mechanistic and black-box deep learning models were both trained on the same set of simulated receptor occupancy time course data as output by the mechanistic model. The semi-mechanistic deep learning model (red) had a lower intial error and final error than its black-box counterpart (blue). (**A**): The models were first compared over the course of 88 epochs (number of epochs completed in 48 h by semi-mechanistic model) with the end error being 0.173 and 3.76 respectively. (**B**): As the black-box model had a shorter run-time per epoch, the comparison was again made with equal total run time (48 h). The reduced final training error of the black box model (2.39) was still more than tenfold that of the semi-mechanistic deep-learning model.

Following Wang et al.^14^, we used the mechanistic model to generate some simulation results to train the neural network models (see Methods). Figure 3 demonstrates the training error comparison between the black-box (blue) and semi-mechanistic deep learning model (red). To compare the training efficiency, both models were trained for the same time period (48 h). The error for the semi-mechanistic model is significantly lower than its black-box counterpart, reaching a training error of 0.17 compared to 2.25 at the end of the training. The training process also converges much more quickly. After approximately 8 h, the semi-mechanistic model’s training error drops to 2, which is not only four-fold lower than the black-box model’s error of 8.7 at the same time point, but also lower than the black-box model’s minimum training error after 48 h. The difference in training error is similarly seen on a per epoch basis. The semi-mechanistic model first outperforms the final training error for the black-box model (there are 192 epochs in the 48 h period) by epoch 15. Exploratory analysis using longer training time indicates that training error for the black-box’s error plateaus above the semi-mechanistic AI minimum error with a significant margin. After 72 h, the black-box model error is above 1.0, more than 5 times the final error for its semi-mechanistic counterpart.

### The semi-mechanistic deep learning model can substitute the mechanistic model for population simulation

One important application of mechanistic models is to simulate large quantities of parameter combinations to represent populations of virtual subjects. For example, the mechanistic PK-PD model in Fig. 1 can be used to answer the question: if a specific population of subjects (defined by a specific kinetic parameter set) received a certain dose of carfentanil to suppress respiration and then a certain dose of naloxone for rescue, what is the median and 95% confidence interval (CI) of the time course of minute ventilation for this population? We generated a population of 2000 virtual subjects not seen in training and used both the semi-mechanistic and black-box deep learning models to answer this question for different opioids and naloxone dosing scenarios (see Methods).

**Figure 4:**
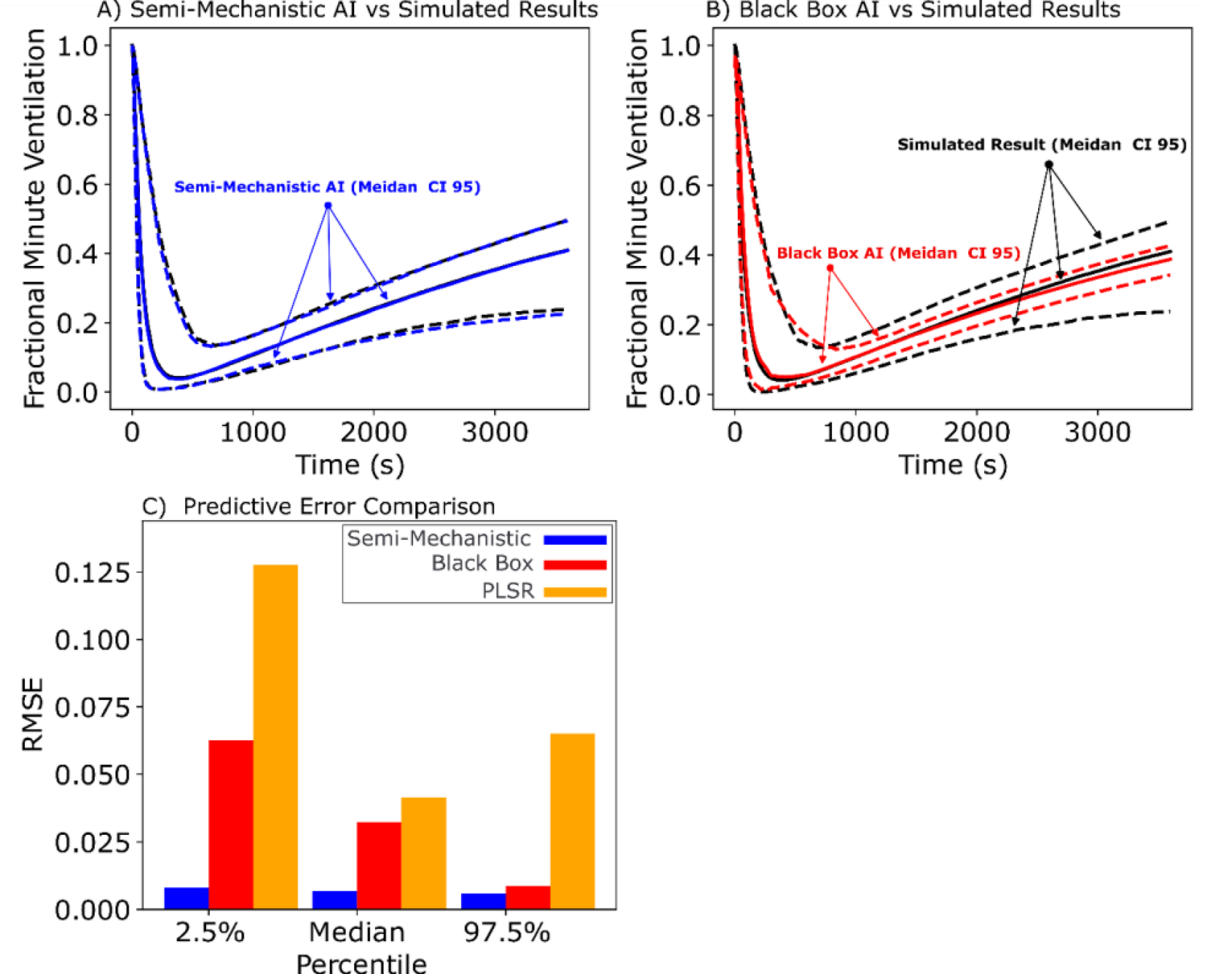
Comparison of Predictive Capabilities for the Semi-mechanistic and Black Box AI models. Both AI models were employed to predict changes in fractional minute ventilation following opioid overdose induced by 0.11 mg carfentanil for a new population of virtual patients (with kinetic parameter combinations not seen during training). (**A**): The semi-mechanistic model (blue) compared against the mechanistic simulation (black). (**B**): The black box AI model (red) compared against the mechanistic simulation (black). Both AI models were able to capture the general trend of median results (solid lines) for the simulated model. However, the semi-mechanistic model’s prediction is identical to the mechanistic simulation (blue and black solid lines superimposed completely) while the black-box model’s prediction is not (red line deviates from the black line in some part). Moreover, the black box model has greater difficulty capturing the level of variability seen in the virtual populations and deviates from the simulated 95% CI much more significantly than the semi-mechanistic model. (**C**): The Root Mean Square Error (RMSE) for the full time course of fractional minute ventilation was calculated for AI models along with Partial Least Squares Regression (PLSR) against the full range of simulated results (540 dosing scenarios for 2000 virtual subjects). RMSE was measured as follows: the median results for the semi-mechanistic, black box, and PLSR models were 0.007, 0.032, and 0.041, respectively. The 2.5% confidence intervals (CIs) were 0.008, 0.062, and 0.128, and the 97.5% CIs were 0.006, 0.009, and 0.065, for each model respectively. The median RMSE for the black box and PLSR models were ~ 5 and ~ 6 times higher, respectively, compared to the semi-mechanistic model. A similar pattern of the semi-mechanistic model outperforming the others was observed at the 2.5% and 97.5% percentiles.

The time course comparison of each of the two deep learning models against the simulation results from the mechanistic model (as the target of emulation) for a specific dosing scenario (carfentanil 0.11 mg intravenous injection, followed by naloxone 4 mg intranasal administration after minute ventilation dropped to 25% of baseline), can be seen in Fig. 4. Both the semi-mechanistic and black-box deep learning models are able to capture the overall trend and the “reversal point” of the median time course of minute ventilation for the virtual population (Fig. 4(A,B)). However, the semi-mechanistic deep learning model is better able to capture the minute ventilation at nadir (lowest point) as well as at the end of the 1 h time course (Fig. 4(A,B)). The difference in performance between the two models becomes more apparent when predicting the 95% CI of the population results. The semi-mechanistic model captures both the 2.5th and 97.5th (Fig. 4(A) blue) of the time course of minute ventilation in the population very well. However, the black-box model misses the time to nadir of the 2.5th percentile time course by approximately 200 s and the inaccuracy is increased for both the 97.5th and 2.5th percentile minute ventilation values near the end of the time course (Fig. 4(B) red).

To quantify the overall performance over all the 540 dosing scenarios (see Methods), we calculated the root mean squared error (RMSE) between the mechanistic model simulation and either the semi-mechanistic or black-box deep learning model predictions. The semi-mechanistic deep learning model had RMSE values of 0.2, 0.375 and 0.35 for the median, 2.5th percentile, and 97.5th percentile time course minute ventilation data, respectively. In comparison, the black-box model had RMSE values of 0.6, 1.27, and 1.37 respectively (Fig. 4(C)). Of note, both the black-box and semi-mechanistic models outperformed a “baseline” method of using PLSR (Partial Least Square Regression) to emulate the mechanistic model^27^.

One key advantage of using a deep learning model to emulate a mechanistic one is the massive acceleration in computational speed. When the number of parameter sets (virtual subjects) is relatively small, for example finishing one single dosing scenario for a population of 2000 virtual subjects, the time taken by the deep learning models is approximately 7 times shorter than the mechanistic model. The speed gain for the deep learning framework increases as the number of simulations increases (more virtual subjects or more dosing scenarios) because the start up time is slower but individual runs are significantly faster. To finish all 540 dosing scenarios for the population, the deep learning models used less than 19 min, while using the mechanistic model to finish all these simulations would take over 10 days (see Methods).

## Discussion

Herein, we presented a machine learning modeling framework designed to improve interpretability of results and alleviate some concerns over the “black box” nature of AI models. The key feature of this model, that improves both end user and researcher comprehension, is that it maintains the mechanistic representation of the underlying physiological processes when emulating a mechanistic model to simulate a target system. While in this work the semi-mechanistic deep learning framework has been applied to a simplified version of our previously published opioid overdose model^15^, the strategy should be applicable to any systems where mechanistic information about internal processes underlying some system dynamics is available.

In addition to being more interpretable, the semi-mechanistic model also shows improvements over its black-box counterpart in both its training and predictive capabilities. From the outset the training error is greatly reduced, with the semi-mechanistic neural networks reaching the minimum error of the black-box neural networks 8 times faster (6 h vs. 48 h) without sacrificing any predictive accuracy. This reduction in training time would further increase the advantage of such a deep learning framework to be used in place of mechanistic models, as now the time cost of “converting” an established mechanistic model to a deep learning emulator is greatly reduced. On the other hand, the fact that the semi-mechanistic deep learning model can achieve a lower training error without overfitting (as evidenced by predicting new data in Fig. 4) suggests that reorganizing the neural networks to mimic the structure of a mechanistic model allows it to learn some information or pattern contained in the target system better than stacking up layers of neural networks (the “black box”).

One specific application we demonstrated using our semi-mechanistic deep learning framework is to use such models (after being adequately trained to emulate a mechanistic model) to predict outcomes from large virtual populations relatively quickly. The speed gain compared to the default method (running the mechanistic model directly) depends on the complexity of the model, the software and hardware used, and the parameter space (number of potential virtual subjects or simulation scenarios). Mechanistic PK-PD models like the one we used in this study most likely would benefit from this approach because these models are complex enough to warrant a semi-mechanistic reorganizing of the deep learning neural networks, and often require the exploration of a large parameter space (e.g., global sensitivity analysis or uncertainty quantification^28,29^) or a large number of scenarios (e.g., the 540 different simulation scenarios used in this work only represent a tiny fraction of all possible combinations of opioids and naloxone dosing schemes).

There is one limitation to the methodology employed in this study when expanding to other translational models. While, in theory, this methodology should be directly applicable to other mechanistic scenarios; it has only been tested and implemented for a simplified version of our translational model to simulate opioid receptor occupancy. Future research will expand this model first to the full translational model simulations^15^ and then to other mechanistic scenarios to confirm this assumption. Similarly, we did not perform a systematic comparison between our AI models and other data-driven models in the context of emulating mechanistic models, such as Partial Least Square Regression (PLSR). Even though one implementation of PLSR was used in Fig. 4(C), it is intended to serve as a “baseline prediction performance” rather than a true evaluation of such methods, given that there are many different variants and improvements of PLSR that we did not implement^27,30,31^.

In summary, we implemented a machine learning framework that maintains the mechanistic structure of its translational model counterpart, allowing us to peer into the “black box” of artificial intelligence modeling and produce interpretable results. This framework can be expanded to cover more complex models, for instance additional opioid scenarios and opioid antagonist formulations^15,32,33^, to leverage its computational efficiency and interpretability to improve understanding of overdose patient outcomes in the community setting. While the concept of reorganizing neural network structures to mimic the target system only applies to those deep learning models that are designed to emulate mechanistic models, this initial effort to “break” the black box can serve as an example for increasing interpretability of other AI-based models across different areas.

## Data Availability

All codes necessary to perform training and prediction using the semi-mechanistic or black-box models are available on the GitHub repository. Additional data, including training data, will be available upon request. Please contact the corresponding author Dr. Zhihua Li (Zhihua.li@fda.hhs.gov).

## Code availability

Codes to perform training and prediction using the semi-mechanistic or black-box models are available in the machine learning branch of the team’s GitHub repository: https://github.com/FDA/Mechanistic-PK-PD-Model-to-Rescue-Opioid-Overdose/blob/Breaking-the-Black-Box-ML/README.md.
